# The Lindbladian form and the reincarnation of Felix Bloch's generalized theory of relaxation

**DOI:** 10.5194/mr-2-689-2021

**Published:** 2021-08-26

**Authors:** Thomas M. Barbara

**Affiliations:** Advanced Imaging Research Center, Oregon Health and Sciences University, Portland, OR 97239, USA

## Abstract

The relationship between the classic magnetic resonance density matrix relaxation theories of Bloch and Hubbard and the modern Lindbladian master equation methods are explored. These classic theories are in full agreement with the latest results obtained by the modern methods. A careful scrutiny shows that this also holds true for Redfield's later treatment, offered in 1965. The early contributions of Bloch and Hubbard to rotating-frame relaxation theory are also highlighted. Taken together, these seminal efforts of Bloch and Hubbard can enjoy a new birth of contemporary relevance in magnetic resonance.

## 1 Introduction

In a recent and important publication, Bengs and Levitt (2019) formalize NMR relaxation theory for systems that deviate significantly from the equilibrium state, which are conditions that invalidate the high-temperature weak-ordering approximation, a corner stone in the commonly used Redfield theory (Redfield, 1957). Bengs and Levitt employed very modern methods that arose in the late 1970s and are now fundamental in the topic of open quantum systems. These methods are often referred to eponymously as the “Lindbladian Form”. In addition to the work of Bengs and Levitt, a useful tutorial on this topic can be found in the work of Manzano (2020). Even though many, if not all, of the classic papers are cited in their offering of their own Lindbladian analysis, Bengs and Levitt were not aware that a result identical to their own was already offered by Bloch (1957) and masterly expounded upon by Hubbard (1961). This conclusion can be ascertained by a glance at Table 1 of (Bengs, 2019), where references to neither Bloch nor Hubbard appear in the rightmost column. From a study of past efforts, as reviewed by Bengs and Levitt along with their own results, this holds for Abragam, for Jeener, and for Ernst. The significance of Bloch and Hubbard has gone unappreciated by the NMR community for decades. By approaching the problem from a new perspective and, importantly, with new experimental measurements on specially prepared spin systems, Bengs and Levitt have resolved a long-standing and long misunderstood issue.

This confusion over the results of Bloch and Hubbard is likely due, in part, to the use of difficult notation by Bloch. Hubbard's treatment is a significant improvement but also possesses a few obscure aspects. In that situation, it is not difficult to understand that nearly all NMR researchers rely on the simpler Redfield formalism, especially given the fact that the conditions under which the approximations are applicable are those encountered in the vast majority of cases. Bengs and Levitt make a detailed comparison between other proposals for a more proper relaxation theory that naturally contains the correct equilibrium steady state, and all are found to be defective in one way or another. One argument for the importance of Bengs and Levitt's effort is their unequivocal and independent confirmation of the success of Bloch and Hubbard over the other formulations. The claim, that a significant aspect of relaxation theory, with an overcast coastline, now enjoys blue sky, is a fair one.

A *brief* exposition of Bloch's main results, by way of Hubbard, using Hubbard's own notation, appears therefore to be a worthwhile endeavor, with historical importance and reasonable expectations that such an effort will be of some interest to NMR researchers in general. How this task has fallen into the hands of the author may also be of some help in orienting the reader.

The origins reside in the author's thesis work, a part of which involved the problem of anisotropic spin–lattice relaxation measurements for deuterated molecules dissolved in a liquid crystalline matrix. This induced the author to study all the early papers on relaxation theory very carefully. Discovering the significance of Bloch, and the clarity of Hubbard's exposition, left an indelible impression, even though the author happily used the simpler Redfield formalism for the task at hand. The author abandoned the field in 1988 to pursue other interests but always enjoyed reading the current NMR relaxation literature. Twenty-five years later, a coworker gently persuaded the author to work out aspects of relaxation and exchange during adiabatic sweeps (Barbara, 2016). After completing that task, the author reacquainted himself with Bloch and Hubbard in order to understand some lingering details. This effort fully prepared the author to recognize how the work of Bloch and Hubbard tied into, and formed a precedent for, the recent efforts of Bengs and Levitt (2019).

The main goal is then to *go beyond a citation* and demonstrate the equivalence of the major result in Bengs and Levitt to that of Bloch and Hubbard. The significant and original result offered here is in the mathematical analysis required to reveal the equivalent success of Bloch and Hubbard towards the problem confronted and solved by Bengs and Levitt by their use of Lindbladian methods. The effort is not trivial, and it requires a careful study of Hubbard's notation and the development of symmetry properties that Hubbard does not provide. Furthermore, Hubbard offers expressions that in some ways are superior to the “fully reduced” Lindbladian form, as will be discussed in Sect. 3. In the conclusion section, the relation of these aspects of relaxation theory to later work by Redfield is commented on.

A discussion of the Lindbladian form is presented beforehand, from a simple and very direct approach which possesses useful didactics, thereby allowing non-experts to appreciate the essence of the formalism without going into a full review of the mathematical details involved in a rigorous proof. Since the Lindbladian approach is rather new to the NMR literature, it can be helpful to have a more facile presentation. Those readers interested in more details can find valuable sources in Bengs and Levitt (2019), Manzano (2020), and Gyamfi (2020).

In addition to the Lindbladian form of Bloch's generalized theory of relaxation, we take advantage of the opportunity to highlight Bloch's and Hubbard's early, and also largely unrecognized, contributions to the dynamics of spin locking and rotating-frame relaxation. Together these aspects form a basis for a renewed interest or, at the very least, a new and greater appreciation of these classic publications.

## 2 A guide to Lindblad

The original publications on the Lindbladian form are of a very mathematical flavor, both for the case of finite dimensions (Gorini et al., 1976) as well as for a general Hilbert space (Lindblad, 1976). Because of these combined efforts, the Lindbladian form is also often referred to as the GKSL equation (Gorini–Kossakowski–Sudarshan–Lindblad). For finite dimensional problems, as is pertinent to NMR applications, it is possible to offer a straightforward method of construction that is reasonably motivated and which uses elementary operator algebra. What follows below is very much ex post facto, and it was developed by the author after reading Bengs and Levitt (2019). It is not without precedent however. In an interesting historical overview of the Lindbladian form (Chruscinski and Pascazio, 2017), one can find examples of nearly identical approaches and even a very early use of the Lindbladian form by Landau in 1927. For a very readable tutorial and overview, Manzano and Gyamfi are recommended. For strictly mathematical proofs on the semi-group, Gorini (1976) and Lindblad (1976) should be consulted. The reader should not interpret what follows as a replacement of the rigorous mathematical proofs of the semi-group. The key to the approach is in the importance of matrix factoring. Indeed, matrix factoring in terms of Kronecker products is an essential ingredient in the mathematical proofs, but its role is not often explicitly highlighted in the manner given here. Matrix products also arise in a straightforward manner when relaxation theory is approached by way of weak coupling perturbation theory for the spin system and the bath, so in this case the spin matrices are already factored. Along the way, the simplest Lindbladian, i.e., the one used for exchange in NMR and which involves the factoring of the identity operator, is presented.

As an ansatz, one can start from a *generalized* “state vector” dynamics, governed by a general complex operator M

1 $$\dot{c_{i}}=\sum_{j}M_{ij}\;c_{j}.$$

In Eq. (1), M generalizes what is usually the Hamiltonian, so the equation, in a purely analogous manner, represents a type of “time-dependent Schrodinger equation” for a finite number of states. From the vector components $c_{i}$ , we construct a Hermitian rank-one “density operator” with elements

2 $$\varrho_{ij}=c_{i}c_{j}^{*}.$$

The square of all rank-one operators are proportional to themselves, $\varrho^{2}=\alpha \varrho$ , so in this case $\alpha =\Sigma c_{i}c_{i}^{*}$ . However, keep in mind that the converse does not hold true. The terminology of “rank” has a number of variants, and in this section we adopt the usage common in the theory of linear vector spaces (Halmos, 1958).

The terms “state vector” and “density operator” have been placed in quotation marks to emphasize their heuristic labels at this early stage of the construction. The dynamics of this “density operator” are given by

3 $$\dot{\varrho }=M\varrho +\;\varrho M^{†}.$$

The solution to Eq. (3) can be expressed as

4 $$\varrho \left(t\right)=\Upsilon \left(t\right)\varrho \left(0\right)\Upsilon (t{)}^{†};\dot{\Upsilon }(t)=M\Upsilon (t);\Upsilon \left(0\right)=1.$$

Of course, Υ(t) is not unitary since M is not necessarily skew Hermitian. After some type of ensemble averaging, the “density operator” will *no longer be rank one* and will take on the character of a general Hermitian operator. This construction is a simple adaptation of the procedure delineated by Landau and Lifschitz (Landau and Lifschitz, 1977). Conservation of probability $\mathrm{Tr}\left(\dot{\varrho }\right)=0$ does not hold for Eq. (3), but we are now just two steps away from rectifying that shortcoming. An application of *Cartesian decomposition* allows any operator to be written in the form

5 $$M=-\frac{1}{2}H+A,$$

where H is Hermitian ( $H=H^{†}$ ) and A is anti-Hermitian ( $A=-A^{†}$ ), which explains why the notation has been used. The reason for the particular numerical factor of -1/2 in front of H will be revealed below. The dynamics can then be expressed in terms of commutators [x,y] and anti-commutators {x,y}

6 $$\dot{\varrho }=-\frac{1}{2}\left\{H,\varrho \right\}+\left[A,\varrho \right]$$

Only the anti-commutator term contributes to $\mathrm{Tr}\left(\dot{\varrho }\right)=-\mathrm{Tr}(H\varrho )$ . If H possesses a non-trivial factoring such that $H=NN^{†}$ , conservation of probability can be non-trivially restored by adding a term in $N^{†}\varrho N$ , due to the cyclic properties of the trace operation $\mathrm{Tr}\left(N^{†}\varrho N\right)=\mathrm{Tr}\left(NN^{†}\varrho \right)$ . The augmented dynamics are then transformed into the famous Lindbladian form:

7 $$\dot{\varrho }=-\frac{1}{2}\left\{NN^{†},\varrho \right\}+\left[A,\varrho \right]+N^{†}\varrho N.$$

The factoring adopted above is known in the numerical matrix analysis literature as Cholesky decomposition (Press et al., 1992) and is closely related to polar decomposition, where an arbitrary linear transformation can be written as a product of a positive matrix and an isometry (Halmos, 1958). While positive, it may not be completely positive. The condition of complete positivity requires that the Kronecker product of the matrix with the identity matrix of arbitrary dimension will also be positive (Manzano, 2020). This condition has been essential for a strict mathematical proof and also ensures that the density matrix has real, positive eigenvalues. Complete positivity has been critiqued in Pechukas (1994) and Shaji and Sudarshan (2005).

According to Eq. (4), the commutator part *and* the anti-commutator part of Eq. (7) can be eliminated from Eq. (7) by the usual method of performing an interaction contact transformation, which then produces a new dynamical equation ${\dot{\varrho }}^{\prime }=kV^{†}\left(t\right)\varrho^{\prime }V\left(t\right)$ . The choice of scaling in Eq. (6) is made so that k= 1. Throughout the steps used to construct Eq. (7), no assumptions regarding the time dependence of the operators are made; therefore, a semi-group structure, where the total propagation must satisfy $T\left(t_{2}+t_{1}\right)=T\left(t_{2})T(t_{1}\right)$ , does not constitute an essential requirement in this *construction* of the Lindbladian form. This provides some evidence that the exercise offered above is something more than merely an effort to cut corners.

It is interesting to compare conservation of probability for the density operator, $\mathrm{Tr}\dot{\varrho }=0$ , with that for pure states, $\frac{d}{dt}\sum c_{i}c_{i}^{*}=0$ . This is a more strict condition and holds if and only if $M^{†}=\;-M$ as is usual for unitary quantum state evolution with conservation of probability where $\sum c_{i}c_{i}^{*}=1$ and M can be written as iH where H is now the Hamiltonian. By incorporating this condition into a higher-dimension structure, we enjoy greater latitude in having Hermiticity and probability conservation along with richer dynamics that can represent relaxation effects.

The anti-Hermitian part of Eq. (5) has a trace-preserving Hamiltonian evolution and can be taken to represent relaxation induced shifts or so-called *dynamic frequency shifts*. These are often small in NMR applications but not exclusively so. A review of these effects in NMR can be found in Weberlow and London (1996), and this aspect will not be pursued further here.

The Lindbladian form is often written as above, but it is important to recognize that it can be expressed in terms of commutators, as was used in the original work by Gorini et al. (1976):

8 $$\left[N,\varrho N^{†}\right]+\left[N\varrho ,N^{†}\right]=-\left\{N^{†}N,\varrho \right\}+2N\varrho N^{†}.$$

In this equation, the alternative factoring, $H=N^{†}N$ , has been adopted. If N is normal, $[N^{†}N]=0$ , the ordering is not important, and in this case, the identity, which corresponds to an infinite temperature limit to within a scalar factor, is a steady state. A similar example is afforded by the factoring given by $H=\left\{H_{1},H_{2}\right\}=\;H_{1}H_{2}+H_{2}H_{1}$ where both operators are Hermitian. The resulting expression can be written in terms of nested commutators:

9 $$\left[\left[H_{1},\varrho \right],H_{2}\right]+\left[\left[H_{2},\varrho \right],H_{1}\right].$$

The anti-commutator can also be re-expressed as a difference between $S^{2}=(H_{1}+H_{2}{)}^{2}$ and $D^{2}=(H_{1}-H_{2}{)}^{2}$ and the above equation can written as the combination

10 $$\left[S\varrho ,S^{†}\right]+[S\varrho S^{†}]-\left(\left[D\varrho ,D^{†}\right]+\left[D,\varrho D^{†}\right]\right).$$

Even though S and D are both Hermitian, Hermitian conjugates have been kept explicit in order to conform with Eq. (8). One simple method for constructing a non-normal factoring is to insert the identity between factors. Write $H=P^{2}=Pe^{iQ}e^{-iQ}P$ with $\left[P,Q\right]\;\neq 0$ so that $N=Pe^{iQ}$ and $N^{†}=e^{-iQ}P$ . Of course, Q must be Hermitian in order to preserve the Hermitian character of the density operator in Eq. (7). Non-normal expansions are also generated quite naturally when spectral decomposition is employed. That procedure will be treated in the next section when the operator expansions of Bloch and Hubbard are discussed.

While a non-trivial factoring excludes the identity matrix as a sole factor, the identity operator itself can often be factored. If H=1=RR the Lindbladian is now the traditional expression used for the description of intramolecular exchange processes in NMR (Alexander, 1962a)

11 $$k\left(R\varrho R-\varrho \right).$$

In Eq. (11), the constant k is now used to denote the rate of exchange. The case of *intermolecular* exchange is considerably more complicated, and it is generally nonlinear, unless high temperatures and small deviations from equilibrium hold forth (Alexander, 1962b). It is worth pointing out here that in the literature on the Lindbladian form the N operators used are often referred to as “jump operators” (Manzano, 2020).

The use of Cartesian decomposition in Eq. (5) is not actually necessary. If M can be factored, for example, as the product $AB^{†}$ , one can directly write down

12 $$\dot{\varrho }=AB^{†}\varrho +\varrho BA^{†}-A^{†}\varrho B-\;B^{†}\varrho A$$

or

13 $$\dot{\varrho }\;=-\left\{\left[B^{†}\varrho ,A\;\right]+\left[A^{†},\varrho B\right]\right\}.$$

This non-Hermitian mixed form is useful when comparing Lindbladian expressions with the early relaxation theories of Bloch and Hubbard. In these, the use of spherical tensor operators can obscure the Hermitian character of certain expressions and also produce expressions that at first blush do not appear to be strictly Lindbladian.

Given that there are $n^{2}-1$ independent operators excluding the identity matrix, we can expect to have a linear combination of Lindbladian forms, with coefficients that are not related in a rank-one fashion, just as with the density operator. These coefficients represent generalized transport parameters.

Whereas this guide to Lindblad should be sufficient for practical purposes, the perspective from a differential approach may not be satisfactory for some readers, perhaps even confusing given the heuristic nature of the construction. Therefore, an outline of the approach from the general solution perspective is offered in the appendix.

The constraints of the Lindbladian form, though powerful, do not provide a complete theory of irreversibility. Other considerations must be brought to bear on the exact manner in which a complicated many-body theory that is fundamentally reversible can be reduced to a simpler, but now apparently irreversible, one. Assumptions of weak coupling between systems (spins and bath) and loss of long timescale correlations as applied to perturbation theory are common elements throughout both modern Lindbladian and the Bloch–Hubbard approaches. Additionally, the need to invoke the secular approximation is paramount to all such approaches, as will be discussed in the next section.

## 3 Bloch–Hubbard relaxation theory and the Lindbladian master equation

As announced in the introduction, it is not the author's goal to give an in-depth review of the Bloch–Hubbard theory. In particular, Hubbard's exposition is especially clear on most accounts, and those with sufficient interest can directly consult the original publications. Rather, the intention is to provide reasons and the motivation for others to read or revisit these classic works. It is useful to point out here at the outset that many of the mathematical methods used with modern Lindbladian approaches to Markovian systems are exactly those used by Bloch (1957) and Hubbard (1961), and other aspects of this fact will be emphasized in the conclusions. Since the success of the Bloch–Hubbard theory has gone unrecognized for so many years – and has now been brought into the limelight by the work of Bengs and Levitt (2019) – a demonstration of the equivalence can be considered an original contribution to the topic.

We can start with Eq. (100) of Hubbard's excellent review article of Bloch's generalized theory:

14 $$\begin{array}{rl} R\left(\sigma \right) & =\sum_{kls}\mathrm{sech}\left(\beta \omega_{s}^{l}/2\right)J_{lk}(\omega_{s}^{l})\{\left[O\left(-\beta \right)V_{s}^{l}O\left(\beta \right)\sigma ,V^{k}\right]\\ & -\left[\sigma O\left(\beta \right)V_{s}^{l}O\left(-\beta \right),V^{k}\right]\}. \end{array}$$

Here we now adopt the notation used by Hubbard, and the reader should keep this change in mind. Hubbard uses σ to denote the spin density matrix. The $V_{s}^{l}$ operators act on the spin states, and a further description of them will be given shortly, as well as how the frequencies $\omega_{s}^{l}$ are determined by the eigenvalues of the spin Hamiltonian E . The operator $O\left(\beta \right)=\mathrm{exp}(\frac{\beta E}{2})$ is related to the equilibrium value of the density matrix. Clearly, $R\left(\sigma \right)$ is linear in σ , and as Hubbard points out, it is easy to see that if the density matrix is at equilibrium, $R\left(\sigma_{\mathrm{eq}}\right)=0$ . The commutator form of Hubbard's equation above is very suggestive. It is almost Lindbladian but not quite the same as the canonical form.

The sums in Eq. (14) are over integer steps from -n to +n for each index, with different values of n for (k,l) and s . The indexed operators and frequencies satisfy symmetries for negative and positive values of their indices:

15 $$(V_{s}^{l}{)}^{†}=V_{-s}^{-l};\omega_{-s}^{-l}=-\omega_{s}^{l};V^{l}=\sum_{s}V_{s}^{l}.$$

In order to manipulate Hubbard's expression, we also need some symmetry properties of the $J_{kl}\left(\omega \right)$ . These index symmetries follow in a straightforward manner from their definitions, which we list for completeness and also adhering to Hubbard's original notation:

16 $$J_{lk}\left(\omega \right)=\frac{1}{2}\int_{-\infty }^{\infty }d\tau C_{lk}\left(\tau \right)e^{i\omega \tau },$$

where

17 $$C_{kl}\left(\tau \right)=\frac{1}{2}\left(A_{kl}\left(\tau \right)+A_{kl}\left(-\tau \right)\right)$$

and

18 $$A_{kl}\left(\tau \right)={\mathrm{Tr}}_{b}\left(\rho^{T}U^{k}(\tau )U^{l}\right)={\mathrm{Tr}}_{b}\left(\rho^{T}U^{k}U^{l}(-\tau )\right).$$

The U operators are bath operators, the ${\mathrm{Tr}}_{b}$ is a partial trace over bath degrees of freedom with bath equilibrium density matrix $\rho^{T}$ , and the time dependence is that given by propagation by the bath Hamiltonian. The index symmetries for the J 's are then

19 $$J_{kl}\left(\omega \right)=\;J_{lk}\left(-\omega \right)=J_{-k-l}^{*}(-\omega ).$$

We can now re-sum Hubbard's expression by way of the substitutions l→-l and s→-s in the first term and k→-k in the second and using

20 $$J_{-lk}\left(\omega_{-l}^{-s}\right)=J_{l-k}^{*}\left(-\omega_{-s}^{-l}\right)=J_{l-k}^{*}\left(\omega_{s}^{l}\right).$$

The commutators in Eq. (14) are then transformed into

21 $$\left[X^{†}\sigma ,V^{k}\right]+\left[{\left(V^{k}\right)}^{†},\sigma X\right],$$

where

22 $$X=\mathrm{sech}\left(\beta \omega_{s}^{l}/2\right)J_{l-k}\left(\omega_{s}^{l}\right)O\left(\beta \right)V_{s}^{l}O\left(-\beta \right).$$

We have transformed Hubbard's expression into Lindbladian form of the “non-Hermitian” type as discussed in Sect. 2. In doing so, the ease in demonstrating $R(\sigma_{\mathrm{eq}})=0$ has been lost but can be restored by combining it with the alternative choice of substitutions k→-k in the first term and l→-l and s→-s in the second term and averaging the two results.

Before reducing Eq. (22) further, an explication of Hubbard's operators is needed. As is common in NMR, the interaction of spin and lattice degrees of freedom are decomposed into products and indexed in the same manner as Hubbard employs. Hermiticity is enforced by stipulating that operators with indices of opposite sign are Hermitian conjugate to each other. The standard spherical tensor operators of rank L and projection m are of this type, and while Hubbard does not explicitly indicate this until examples are offered at the end of his article, his $V^{k}$ operators are basically spherical tensors, where Hubbard uses k to denote the projection index m and suppresses the rank index L . Likewise, Hubbard is not very explicit regarding his $V_{s}^{l}$ operators. He gives their desired properties but not much on a *general* method for their construction. The key idea is that of spectral decomposition (Halmos, 1958), which produces an operator expansion whose coefficients are the eigenvalues of the operator. For the Hamiltonian E we have

23 $$E=\sum_{i}\omega_{i}E_{i},$$

where $\omega_{i}$ represents the eigenvalues of E , and the $E_{i}$ represents projection operators with the properties

24 $$E_{i}E_{j}=\delta_{ij}E_{j};\mathrm{Tr}\left(E_{i}E_{j}\right)=\delta_{ij};\sum_{i}E_{i}=1.$$

There are a number of methods for constructing the projectors. Perhaps the most straightforward is to use the unitary operator, U , which brings the operator E to diagonal form. With U at hand we have

25 $$E_{i}=UX^{ii}U^{†}.$$

The fundamental basis matrices have elements $(X^{ij}{)}_{\alpha \beta }=\delta_{i\alpha }\delta_{j\beta }$ . A family of matrices can now be constructed from a starting operator V which will be “eigen-operators” of the Hamiltonian propagator:

26E,EiVEj=ωi-ωjEiVEj,27eiEtEiVEje-iEt=eiωi-ωjtEiVEj.

When the V matrices are defined in the eigenbasis of E , this result is almost trivial, for in that case

28 $$(e^{iEt}Ve^{-iEt}{)}_{ij}=e^{i\left(\omega_{i}-\omega_{j}\right)t}V_{ij}.$$

Even so, the use of projectors allows one to avoid writing out explicit matrix elements. Lexigraphical ordering of these (i,j) index pairs can be adopted and assigned to indices that range from negative to positive integers or odd half integers to obtain Hubbard's $V_{s}^{l}$ and his frequencies $\omega_{s}^{l}$ . The original mathematical lemmas and theorems of Gorini et al. (1976) heavily rely on the use of spectral decomposition. Redfield's notation adopts the use of explicit matrix elements, and this perhaps is another of the reasons for the popularity of his equations. Bengs and Levitt (2019) commence their own analysis by adopting an eigenbasis for E as with Eq. (28). We can now also tie spectral decomposition to the factoring problem of the previous section. If H is a positive operator, we can apply Eq. (23) to decompose H into a sum

29 $$H=\sum_{k}N_{k}N_{k}^{†},$$

where the operators can be written in terms of the unitary operator T which diagonalizes H with positive eigenvalues $\lambda_{k}$ as

30 $$N_{k}=\sqrt{\lambda_{k}}T\;X^{kk}.$$

Returning to Hubbard's relaxation expression, one can use the properties of the $V_{s}^{l}$ to evaluate the effects of the operator O(β) on $V_{s}^{l}$ by employing Eq. (27) with β replacing *it*. We can also expand $V^{k}$ in terms of $V_{s}^{l}$ . Finally, we invoke the secular approximation, where rapidly oscillating terms, generated by the evolution of E , are dropped. If the Zeeman energy is dominant, the spectral decomposition is not needed, since the spherical tensor operators are already eigen-operators. A single sum over the $V^{l}$ terms remains. Otherwise, one needs sums over both l and s :

31 $$\begin{array}{rl} R\left(\sigma \right) & =\sum_{ls}e^{\frac{\beta \omega_{s}^{l}}{2}}J_{l-l}(\omega_{s}^{l})\mathrm{sech}\left(\beta \omega_{s}^{l}/2\right)\\ & \left\{\left[(V_{s}^{l}{)}^{†}\sigma ,V_{s}^{l}\;\right]-[\sigma V_{s}^{l},(V_{s}^{l}{)}^{†}\;]\right\}. \end{array}$$

The double sum in Eq. (31) is useful in zero field. We have now fully reduced Hubbard to Lindbladian form and essentially reproduced the main result obtained by Bengs and Levitt (2019) by way of the Lindbladian formalism. One small difference is Hubbard's use of the thermal symmetrizing factor given by the hyperbolic secant function. If so desired, this can be removed as illustrated in Hubbard's paper. It is also imperative to emphasize the importance of the secular approximation in obtaining the Lindbladian form. Without this step, relaxation in the rotating frame will be time dependent, with very different and perhaps even unphysical dynamics. This same approximation is required in a Lindbladian approach, which is often referred to as the “rotating-wave” approximation (Manzano, 2020).

While very compact, the presence of a finite temperature steady state is definitely obscure. From Eq. (31) directly, the only apparent recourse is to expand the dynamics in a complete set of basis matrices and search for one or more zero eigenvalues. Such a procedure is illustrated in an example with a simple two-dimensional density matrix dynamics in Manzano (2020) where eigenvalues are easily computed. This is in contrast to Hubbard's original expression, Eq. (14), where the steady state is clearly recognizable, even for arbitrarily large dimensions. Alternatively, we can invoke the secular approximation directly to Eq. (14), and we can enjoy a compromise where one retains the clear presence of the steady state. Hubbard (1961) teaches us how to retain explicit information on the fixed-point density matrix. However, this seems to be possible only when dynamic frequency shifts can be ignored.

One should also appreciate that a homogeneous system which possess a zero eigenvalue is closely related to an inhomogeneous system. The procedure of homogenizing an inhomogeneous system by incorporating the inhomogeneous vector into equations with an additional dimension, which is invariant with an eigenvalue of zero, has been employed in a Bloch equation analysis of spin echoes (Bain et al., 2011), steady state precession (Nazarova and Hemminga, 2004), and relaxation (Levitt and Di Bari, 1992). Going in the opposite direction can be considerably more difficult.

## 4 Bloch and Hubbard and rotating-frame relaxation

We now take a side turn to another aspect of the pioneering work of Bloch (1957) and Hubbard (1961), which also has largely gone unrecognized in the NMR literature. As explained in the introduction, the author was recently reacquainted with these aspects in an effort to go beyond a Bloch equation picture with only $R_{1}$ and $R_{2}$ for spin locks and adiabatic sweeps in the presence of exchange (Barbara, 2016). Both examples illustrate the use of the high-temperature weak-ordering situation that occurs when the full theory contained in Eq. (31) is reduced to the appropriate limit for those circumstances. These applications do not require the Lindbladian form. Nevertheless, it strikes the author as a wasted opportunity to not mention the treatment of rotating-frame relaxation by Bloch and Hubbard and therefore reintroduce these two results to 21st century NMR scientists.

At the end of Bloch's paper, he applies his theory to relaxation in the presence of an RF (radiofrequency) field. For a rank-one tensor interaction, such as the fluctuating field relaxation mechanism, he derives a set of generalized Bloch equations in the *rotating frame:*

32 $$\begin{array}{rl} \left(\begin{array}{l} {\dot{M}}_{x}\\ {\dot{M}}_{y}\\ {\dot{M}}_{z} \end{array}\right) & +\left(\begin{array}{lll} A_{x} & -\Omega \mathrm{cos}(\theta ) & a_{x}\\ \Omega \mathrm{sin}(\theta ) & A_{y} & -\Omega \mathrm{sin}(\theta )\\ a_{z} & \Omega \mathrm{sin}(\theta ) & A_{z} \end{array}\right)\\ & \left(\begin{array}{l} M_{x}\\ M_{y}\\ M_{z} \end{array}\right)=\left(\begin{array}{l} c_{x}\\ 0\\ c_{z} \end{array}\right). \end{array}$$

When the Rabi frequency, Ωsin⁡(θ) , is much smaller than the Larmor frequency, $\omega_{0}$ – but still comparable to the resonance offset, Ωcos⁡(θ) – we have $a_{z}=c_{x}=0$ . At high temperature, $c_{z}=R_{1}M_{0}$ as usual. In terms of the spectral densities, $J_{n}\left(\omega \right)$ , the relaxation parameters are given by the equations

33Ax=Ay=J1ω0+J0Ω+J00-J0Ωcos⁡2θ34Az=2J1ω035ax=-J00-J0Ωsin⁡θcos⁡θ.

In the absence of an RF field, θ=0 , and $A_{x}$ and $A_{z}$ are $R_{2}$ and $R_{1}$ respectively. Note that $a_{x}$ is generally not zero if the locking field is off resonance.

It is not always appreciated that the usual formulas for rotating-frame relaxation are those for the case when the locking field, whose magnitude is given by Ω , is much larger that the relaxation rates. In that situation, a first-order perturbation is applicable (Barbara, 2016). If the transformation, denoted by V , diagonalizes the Bloch equations without relaxation, the first-order contribution from the relaxation matrix elements is given by the diagonal elements of $V^{-1}RV$ , which are then rotating-frame relaxation rate constants:

36ρ1=R1cos⁡2θ+R2-J00-J0Ωsin⁡2θ,37ρ2=12R2+12R2cos⁡2θ+R1+J0Ω-J00sin⁡2θ.

When the low frequency terms are collected, one obtains expressions that reproduce those for chemical shift exchange, as usually derived from an analysis of exchange perturbation in the limit of fast exchange using the Bloch–McConnell equations (Barbara, 2016; Abergel and Palmer, 2003). This result is often attributed to Wennerstrom (1972). The theory was already presented in Bloch's paper in 1957. Uncorrelated local fields for two spin 1/2 systems is an important mechanism for spin isomer conversion, as discussed in Bengs and Levitt (2019).

Bloch (1957) presents other applications to his formalism that are of interest. These applications offer an excellent catalytic motivation for going through many of his notational details.

After his own exposition and refinement of Bloch's theory, Hubbard also gives an application to rotating-frame relaxation and ups the ante by considering second rank, dipole–dipole relaxation mechanisms. Hubbard (1961) obtains equations for the magnetization dynamics similar to Eq. (32), with off-diagonal relaxation elements. After taking the first-order contribution, the rotating-frame spin–lattice rate constant is given by

38 $$\begin{array}{rl} \rho_{1} & =\;R_{1}{\mathrm{cos}}^{2}\left(\theta \right)+\;R_{2}{\mathrm{sin}}^{2}\left(\theta \right)\\ & -6{\mathrm{sin}}^{2}\left(\theta \right)\left\{-J_{0}\left(0\right)+{\mathrm{cos}}^{2}\left(\theta \right)J_{0}\left(\Omega \right)+{\mathrm{sin}}^{2}\left(\theta \right)J_{0}\left(2\Omega \right)\right\}. \end{array}$$

In terms of the spectral densities, $R_{1}$ and $R_{2}$ are

39R1=4J1ω0+4J22ω0,40R2=6J00+10J1ω0+4J22ω0.

This result was produced in very different notation in Blicharski (1972). Unfortunately, in that work, the various contributions are gathered together in such a manner as to obscure the origin of and the relationship between each. The reader should keep in mind that for both examples, details regarding scale factors of the spectral densities have been suppressed. These can be added according to the specific needs of their application, be it dipolar, quadrupolar, fluctuating field, or chemical shift exchange.

## 5 Comments and conclusions

Given the maturity of the topic of relaxation in magnetic resonance, it is not often that a surprise is forthcoming. Many modern treatments, that are very application oriented, reflect this maturity. For example, the extensive overview offered by Kowalewski and Maler (Kowalewski and Maler, 2006) details many of the modern applications. Bloch is not listed in the index, and Hubbard is indexed only in the context of work he did on rotational diffusion applications and the calculation of correlation functions, even though Hubbard's review article is cited in the chapter titled “Redfield Relaxation Theory”. In Redfield's later effort, which appeared as a chapter in *Advances in Magnetic Resonance* (Redfield, 1965), Redfield acknowledges the influence of Bloch and offers his own equations that account for relaxation at finite temperatures while only citing Hubbard's review article in passing. Redfield makes no effort to demonstrate or expound on the relationship between his expression and Bloch's or Hubbard's. A glance at Redfield's Eq. (3.15) in Redfield (1965) induces one to question in what way his result is also Lindbladian, for the expression is very different than Hubbard's equation Eq. (100). The factoring of the spectral densities that Hubbard achieves does not rely on the secular approximation. Nonetheless, a careful study reveals that Redfield's equation is also a mixed Lindbladian type, similar to Eq. (21)–(22). Here one can fully appreciate the power of using spectral decomposition to factor out the spectral densities and in doing so produce an expansion in non-Hermitian operators. Redfield's 1965 result, which *is based on a Hermitian operator expansion* and looks nothing like a Lindbladian, is nonetheless as serviceable as Hubbard's.

Approaches to NMR relaxation theory have changed over its history. In the work of Bloch, Redfield, and Hubbard, extensive manipulations are carried out at the level of second-order perturbation theory for the *solutions* to the interaction representation density matrix. At the end of this effort, a finite time step expression is produced, which is argued to be basically the solution to a given differential equation. However, already in the same year that Hubbard's review article appeared in print, Abragam took the alternative approach by directly iterating the differential equation in his treatment of relaxation (Abragam, 1983). This is now the usual practice and is the path taken, for example, by Goldman in his review of NMR relaxation theory (Goldman, 2001), who offers his own treatment of a finite temperature relaxation theory therein and also uses spectral decomposition for that case. This same iteration approach is also adopted by recent expositions using the Lindbladian formalism and weak collision, Markovian bath dynamics, and the secular approximation. Again, a good illustration of this is given in Manzano (2020). As mentioned earlier, a study of that overview reveals that many of the same tools, e.g., use of Hamiltonian projection operators to obtain eigen-matrices, as used by Bloch (1957) and Hubbard (1961), are also brought to bear in the same manner. The historical overview mentioned in Sect. 2 (Chruscinski and Pascazio, 2017) also outlines other open quantum system efforts made by various researchers, and there is a strong enough similarity to suspect that these have rediscovered the main results of Bloch and Hubbard, as well as having anticipated the Lindbladian form.

It is possible to make the argument that NMR theory needs to modernize, in keeping with new approaches that appear to have a more firm foundation in quantum theory. The author is reminded of a classic collection of essays (Peierls, 1979) with the surprise here, that these new methods can find their own perfect reflection in the best work of the old masters.

## Data Availability

No data sets were used in this article.

## Appendix A Some elaborations on Lindblad and Sect. 2

In this appendix, an outline of further aspects of the Lindbladian form is offered. It is self-contained and does not invoke the various mathematical theorems often used as a starting point (Manzano, 2020).

The most general linear transformation for a matrix, in particular the density matrix, can be written in the form

A1
$$\rho^{\prime }=\sum_{\alpha \beta \alpha^{\prime }\beta^{\prime }}C_{\alpha \beta \alpha^{\prime }\beta^{\prime }}\;X^{\alpha \beta }\rho X^{\alpha^{\prime }\beta^{\prime }},$$

where the $X^{\alpha \beta }$ represents the fundamental basis matrices defined in Sect. 3. Rather than derive this equation, one can grasp that it is correct by reducing it to component form

A2
$${\rho_{ij}}^{\prime }=\sum_{\beta \alpha^{\prime }}C_{i\beta \alpha^{\prime }j}\rho_{\beta \alpha^{\prime }}.$$

Here, one recognizes the Redfield notation but with slightly rearranged indices. Introducing a complete set of Hermitian matrices $O_{k}$ provides for a more compact expression where now

A3
$$\rho^{\prime }=\sum_{kk^{\prime }}G_{kk^{\prime }}O_{k}\rho O_{k^{\prime }}.$$

If the transformation preserves the Hermitian character of the density matrix, G is Hermitian in the k indices $G_{kk^{\prime }}={G_{k^{\prime }k}}^{*}$ . If the trace is also invariant, we have

A4
$$\sum_{kk^{\prime }}G_{kk^{\prime }}O_{k^{\prime }}O_{k}=1.$$

This is all we need to obtain a difference equation in $\rho^{\prime }-\rho$ and obtain the Lindbladian form. However this is not usually the way the problem is approached (Manzano, 2020). Instead the starting point is from the “Kraus form”, which is obtained by assuming that the matrix G is positive. Being positive, one can factor G in the same manner illustrated in Sect. 3 via the spectral decomposition for H . This then allows summations over the k indices to produce the Kraus operators for the transformation. The one remaining index is over the eigenvalue index. This is basically going a step too far, and it is more direct to start from Eq. (A3). The required subtraction can be implemented by substituting Eq. (A4) for the identity in a symmetrical manner:

A5
$$\rho^{\prime }-\rho =\sum_{kk^{\prime }}G_{kk^{\prime }}O_{k}\rho O_{k^{\prime }}-\frac{1}{2}\left(\rho 1+1\rho \right).$$

To complete the process, one now extracts those terms in the operator expansion that involves the identity operator, which we ascribe to the zero index $O_{0}=1$ . It is a simple matter to see that these can be collected into the expression

A6
$$\sum_{k}\frac{1}{2i}\left(G_{k0}-{G_{k0}}^{*}\right)i\left[O_{k},\rho \right].$$

This represents the part of the transformation generated by a commutator. The remaining part, where the sums now exclude the identity matrix, is now of the Lindbladian form

A7
$$\rho^{\prime }-\rho =\sum_{kk^{\prime }}G_{kk^{\prime }}\left[O_{k}\rho O_{k^{\prime }}-\frac{1}{2}\left\{\rho ,O_{k^{\prime }}O_{k}\;\right\}\right].$$

One can then use the positivity of G to factor this expression into non-Hermitian operators. In this way one can see that the approaches from the generalized differential equation and the one based on the general solution are equivalent.
